# Artificial Intelligence-Based Approach for Automated Gonad Volume Quantification Using Magnetic Resonance Imaging in Healthy Adolescents Across Puberty

**DOI:** 10.3390/diagnostics16091357

**Published:** 2026-04-30

**Authors:** Fahmida Haque, Stephanie A. Harmon, Allison Kumnick, Mary Soliman, Karen F. Berman, Jack A. Yanovski, Evrim B. Turkbey, Lynnette K. Nieman, Veronica Gomez-Lobo, Shau-Ming Wei, Peter J. Schmidt, Baris Turkbey

**Affiliations:** 1 Artificial Intelligence Resource, National Cancer Institute, National Institute of Health, Bethesda, MD 20892, USA; fahmida.haque@nih.gov (F.H.); stephanie.harmon@nih.gov (S.A.H.); 2 Molecular Imaging Branch, National Cancer Institute, National Institute of Health, Bethesda, MD 20892, USA; 3 Pediatric and Adolescent Gynecology, National Institute of Child Health and Human Development, National Institute of Health, Bethesda, MD 20892, USA; allison.kumnick@nih.gov (A.K.); marsoliman05@gmail.com (M.S.); veronica.gomez-lobo@nih.gov (V.G.-L.); 4 Section on Integrative Neuroimaging, Clinical and Translational Neuroscience Branch, National Institute of Health, Bethesda, MD 20892, USA; bermank@mail.nih.gov; 5 Section on Growth and Obesity, Division of Intramural Research, Eunice Kennedy Shriver National Institute of Child Health and Human Development, National Institute of Health, Bethesda, MD 20892, USA; yanovskj@mail.nih.gov; 6 Radiology and Imaging Sciences Department, National Institute of Health, Bethesda, MD 20892, USA; evrim.turkbey@nih.gov; 7 Diabetes, Endocrinology, and Obesity Branch, National Institute of Diabetes and Digestive and Kidney Diseases, National Institute of Health, Bethesda, MD 20892, USA; niemanl@mail.nih.gov; 8 Section on Behavioral Endocrinology, National Institute of Mental Health, National Institute of Health, Bethesda, MD 20892, USA; shauming@mail.nih.gov (S.-M.W.); peterschmidt@mail.nih.gov (P.J.S.)

**Keywords:** MRI, puberty, adolescents, gonads, artificial intelligence

## Abstract

**Background/Objectives:** MRI is a non-invasive tool which can be used to assess baseline gonadal anatomy, including changes during puberty. Volumetric characterization offers valuable insights about the reproductive system and gonads, but annotation is cumbersome, and no AI tool is currently available. This study aimed to develop two open-source AI models to segment bilateral gonads at MRI scans in healthy subjects. **Materials and Methods:** This study uses a longitudinal dataset consisting of 182 MRIs from 22 healthy girls (median age 13) and 266 MRIs from 44 healthy boys (median age 13) from a single institute. MRI acquisition included T2-weighted (T2W) sequence, along with fat-saturated (FS) T2W when indicated. An expert radiologist segmented gonadal anatomy, including ovarian cysts (>3 cm). Three-dimensional nnUnet models were trained for ovary, cyst, and testicle segmentation, respectively. The ovary–cyst segmentation model was applied to an external dataset with 30 adult subjects. Model performance was evaluated on the test set using the Dice similarity coefficient for ovary (DSCOV), cyst (DSCCY), and testicles (DSCTS). Subject-level total volumes for ovaries (TOV), cysts (TCV), and testicles (TTV) were computed. **Results:** Ovary, cyst, and testicle segmentation models achieved DSCOV of 0.86, DSCCY of 0.69, and DSCTS of 0.90 in the in-house test set, respectively. Average mean difference with 95% confidence intervals for TOV, TCV, and TTV were 0.87 (−5.78, 7.5), −0.41 (−3.3, 2.5), and 0.19 (−1.5, 1.9) cm^3^, respectively. **Conclusions:** The developed models show promising and reliable performance in volumetric and morphologic evaluation of gonads during puberty.

## 1. Introduction

Puberty marks one of the most transformative phases of human development—a complex biological transition towards adult secondary sexual characteristics and reproductive capacity [1]. As the primary reproductive organs, the gonads play a vital role during this process [2]. During puberty, humans go through gonadarche, which involves the growth and functional maturation of the gonads, leading to increased production of sex hormones, the beginning of ovulation and follicle development in females, and sperm production in males [1]. Gonadal growth is a recognized structural marker of pubertal maturation, with testicular volume reflecting Sertoli cell proliferation and spermatogenic potential, and ovarian volume correlating with follicular development and endocrine activity [3,4]. Deviations in gonadal growth trajectories are clinically relevant in the evaluation of delayed or precocious puberty and may have implications for future fertility and reproductive health [3,4]. Therefore, gonadal growth observation is important for puberty, fertility, and adolescence-related studies [3,4].

In clinical practice, medical imaging of the gonads is used for various purposes, e.g., to detect and stage testicular and ovarian cancer, to assess fertility, and common benign conditions such as polycystic ovary syndrome (PCOS) [5,6,7,8]. Ultrasound (US) is the first-line imaging modality for evaluation of the testicles and ovaries in pediatric and adolescent populations due to its accessibility, lack of ionizing radiation, and high-resolution assessment of superficial structures such as the testicles using high-frequency linear transducers [9]. For ovarian imaging, transabdominal US performed with a distended urinary bladder is the standard initial approach; however, image quality may be influenced by factors such as body habitus, bowel gas, and operator dependency, particularly when acoustic windows are suboptimal [10,11]. Although scrotal ultrasound provides a high-resolution evaluation of intratesticular pathology, it has limitations related to operator dependency and clinical expertise, its relatively limited field of view beyond the scrotal sac, and its constrained ability for comprehensive tissue characterization in complex or indeterminate lesions [10,12]. In such settings, magnetic resonance imaging (MRI) serves as a complementary modality when ultrasound findings are inconclusive, offering multiplanar imaging, a larger field of view, and high soft-tissue contrast that reduce operator dependency and acoustic window limitations while enabling comprehensive anatomical and tissue characterization of gonadal organ and pelvic lesions, including physiologic and non-physiologic cysts with reported diagnostic accuracies ranging from 88% to 93% [8,11,13,14,15]. T2-weighted (T2W) MRI provides detailed anatomical delineation of gonadal structures, while fat-saturated T2-weighted (FS T2W) sequences enhance visualization of fluid-containing structures such as ovarian cysts by suppressing background fat signal, thereby improving tissue contrast and lesion conspicuity [8,16].

In the clinical setting for accurate gonadal volume estimation, experts need to manually outline the testicles, ovary, and ovarian cysts on MRI, which is a very time-consuming and laborious process. In recent years, the application of artificial intelligence (AI) for the automated detection and segmentation of organs and lesions in medical imaging has grown substantially, driven by its demonstrated reliability and its potential to reduce the workload of medical imaging physicians [17,18,19]. Due to a lack of expert-annotated scans, there are no publicly available AI-based tools to segment gonads at MRI for adolescents. To develop such an AI algorithm that provides diagnostic information throughout puberty, the training population should include healthy pre- and post-puberty subjects. In this retrospective study, we aim to develop AI models for gonadal segmentation in pelvic MRIs, spanning pre-to-post pubescent individuals.

## 2. Materials and Methods

### 2.1. Study Population

In this retrospective study, subjects from a longitudinal clinical trial investigating endocrine changes during puberty in healthy adolescents (NCT01434368) [20] were used. In this trial, gonadal MRI examinations were performed every 8–10 months beginning at either 8 or 12 years of age at a single institution. Details of the subject protocol and inclusion and exclusion criteria have been discussed in a previous publication [20]. The dataset was collected at a single center. For the ovary and cyst (>3 cm) segmentation AI model development, 182 paired T2W and FS T2W MRI scans from 22 healthy girls (median age 13, range from 8 to 17 years) were used. The median number of scans for healthy girl cohort was 15 (range 6, 26) for T2W and 11 (range 5, 22) for FS T2W scans (Table 1). Based on the Ovarian-Adnexal Reporting and Data System (O-RADS) MRI Committee guideline, ovarian cysts, including follicles, hemorrhagic cysts, and corpus luteal cysts measuring less than 3 cm, are considered normal physiologic observations on MRI [21]. Therefore, we considered cysts with a size greater than 3 cm in our study, which are commonly used for further observation in clinical practice [21,22]. For the development of the testicle segmentation AI model, 266 T2W MRIs from 44 healthy boys (median age 13, range from 8 to 17 years) were used; 3 scans from 2 subjects showed retractile right testicles at an earlier age. The median number of scans for the healthy boy cohort was 6 (range 1, 12) T2W scans (Table 1). In Supplementary Figure S1, details about subject inclusion and exclusion criteria for final cohort selection for AI development are shown.

### 2.2. Image Acquisition and Processing

MRI scans were obtained on a 3 Tesla scanner (Achieva, Philips Medical System) using a phased array surface coil. Imaging pulse sequences included axial and sagittal T2 turbo spin echo (TSE) and axial fat-saturated (FS) weighted MRI. Table 1 presents detailed information about Image acquisition parameters.

An expert radiologist (>20 years of body imaging experience) annotated testicles on T2W MRI and ovaries, and cysts (>3 cm) on FS T2W MRI. The expert radiologist annotated the ovaries and cysts on FS T2W MRI, as they are better visible on FS T2W rather than T2W MRI. On the other hand, testicles are well visible on T2W MRI. Manual segmentations were performed using ITK-SNAP (version 3.6.0; Penn Image Computing and Science Laboratory, University of Pennsylvania, Philadelphia, PA, USA), an open-source software application for three-dimensional medical image visualization and segmentation [23]. Gonadal structures were delineated slice-by-slice in the axial plane annotation tools, with multiplanar views available for anatomical reference. For ovary and cysts annotation, ovaries are marked as label 1, and cysts are marked as label 2. For bilateral testicular annotations, the right-sided testicle is marked as 1, and the left-sided testicle is marked as 2. Expert annotation has been considered as ground truth (GT) in AI development and model evaluation. Before preparing the dataset for the AI model development, the DICOM data were converted into the 3D Neuroimaging Informatics Technology Initiative (NIfTI) format using publicly available methods [24].

### 2.3. Ovary and Ovarian Cyst Segmentation AI Model Development

For ovary and ovarian cyst segmentation AI development, 184 scans from 22 subjects were split into 129, 19, and 36 scans, based on the patient-level stratification for training, validation, and test sets, using a 70:10:20 ratio, respectively. Subjects with multiple scans were put into one of the sets (train, test, or validation) to make sure none were present in two sets. This was done to avoid overfitting the model and remove patient-level biases. The test set was reserved for model evaluation. Supplementary Table S1 shows the total number of scans for train, validation, and test splits with patient-level stratification.

For model development, both T2W and FS T2W MRI sequences were utilized in both multi-modal and single-modality configurations. This design was intended to evaluate the relative performance of each modality in detecting the ovary and ovarian cysts. Because T2W and FS T2W images differ in spatial resolution and voxel spacing, multiple resampling strategies were implemented to systematically assess the impact of resampling on model performance and to identify an optimal approach. T2W MRIs were resampled to the corresponding FS T2W MRI size and spacing and denoted as T2W_FS_, and similarly, FS T2W MRI and expert-annotated masks were resampled to T2W size and spacing and denoted as FS_T2W_. Resampling was applied in both directions to systematically evaluate the effect of alignment strategy on model performance and to determine which approach yields superior performance in downstream tasks.

**Figure 1:**
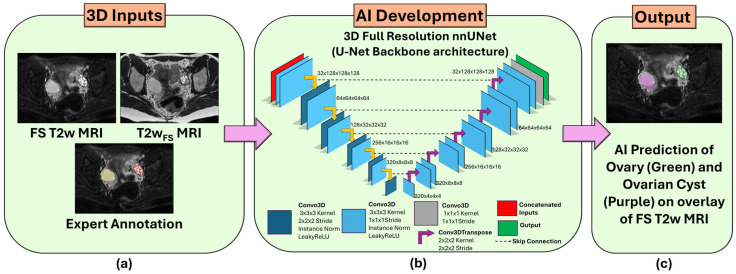
Overview of the overall workflow of the AI model for automated ovary and ovarian cysts segmentation from T2W and FS T2W MRI. (**a**) 3D T2W_FS_ and FS T2W MRI with corresponding expert annotation (ovary in red, ovarian cyst in yellow) were used as inputs for the multi-modality model development. (**b**) 3D full-resolution configuration with U-Net as backbone architecture from the nnUNet framework was trained. (**c**) AI-predicted ovary and ovarian cyst annotations are shown on the FS T2W MRI overlay.

Figure 1 shows the overall workflow of the AI model for automated ovary and ovarian cysts segmentation from T2W and FS T2W MRI. The 3D full-resolution (3D-FullRes) configuration from the nnUNet framework [25] with 3D U-Net [26] as background architecture was utilized. This framework provides an automated pipeline that includes data pre-processing, augmentation, post-processing, and model hyperparameter tuning for optimization to a specific dataset.

Before model training, all MRI volumes underwent standardized pre-processing. To ensure spatial consistency across modalities, input images were resampled to a target spacing of 0.75 × 0.75 × 2.2 mm^3^, reflecting the median dataset resolution, and Z-score normalization was applied independently per modality. Cubic interpolation (order 3) was used for image resampling, while linear interpolation (order 1) was applied to segmentation masks. The 3D full-resolution model was implemented using the nnUNet framework with a PlainConvUNet architecture comprising six stages.

The model was trained to be one-fold due to data limitations. The model was trained for 1000 epochs, with each epoch comprising 250 training iterations. Training was performed with a patch size of 224 × 224 × 40 and a batch size of 2 to accommodate GPU memory constraints. As nnUnet automatically tune hyperparameters according to the dataset, no further hyperparameter tuning was performed [25]. The training began with an initial learning rate of 0.01, which was progressively reduced following the “poly” learning rate schedule, calculated using the formula (1 − epoch/epoch_max_)^0.9^ [27]. The final model was chosen based on the checkpoint that achieved the highest average foreground Dice similarity coefficient during training [28,29]. For evaluation, inference on the test dataset was conducted using a sliding window method with a consistent patch size of 40 × 224 × 224.

We trained the ovary and ovarian cyst segmentation models with single and both MRI sequences. The model trained with only 3D FS T2W MRI was denoted as the FS model. The model trained with only 3D T2W MRI was denoted as the T2W model. The model trained with resampled 3D T2WFS and original 3D FS T2W MRI was denoted as the T2WFS + FS model. The model trained with resampled FST2W and original T2W MRI was denoted as the T2W + FST2W model. So finally, these 4 models were further evaluated to identify the best-performing model.

### 2.4. Testicle Segmentation AI Model Development

**Figure 2:**
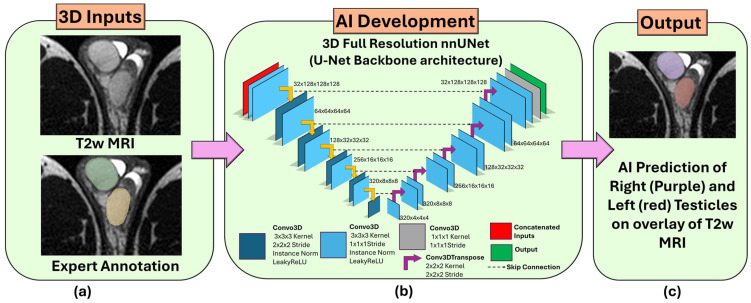
Overview of the overall workflow of the AI model for automated left and right testicle segmentation from T2W MRI. (**a**) 3D T2W MRI with corresponding expert annotation (right testicles in green, left testicles in yellow) were used as inputs for the model development. (**b**) 3D full-resolution configuration with U-Net as backbone architecture from the nnUNet framework was trained. (**c**) AI-predicted left and right testicle annotations are shown on the T2W MRI overlay.

For the testicle segmentation AI development, 266 scans from 44 subjects were split into 180, 27, and 59 scans on the patient-level for training, validation, and test sets, respectively. The two subjects with retractile right testicles were intentionally put into the test set, as this is not a common clinical condition [30] and using these scans in training can mislead the AI model. Furthermore, the dataset was split into patient and age-stratified so that the model could learn from all age distributions (8 to 17) without any patient-level biases and avoid overfitting the model to a specific age and patient. Supplementary Table S2 shows the total number of scans for train, validation, and test splits with patient and age level stratification. For the testicle segmentation model, we trained using only a single MRI sequence (T2W MRI), as T2W MRI has better resolution in capturing testicles compared to FS T2W MRI. Figure 2 shows the testicle segmentation AI development from T2W MRI.

Prior to model training, T2W MRI volumes underwent standardized pre-processing. Images were resampled to a consistent voxel spacing of approximately 0.6 × 0.6 × 2.2 mm^3^, corresponding to the median dataset resolution, to ensure spatial uniformity across subjects. Intensity normalization was performed using Z-score normalization. Cubic interpolation (order 3) was applied for image resampling, and linear interpolation (order 1) was used for segmentation masks.

For model development, we employed the 3D full-resolution (3D_FullRes) configuration within the nnUNet framework [25] along with a modified variant in which the standard U-Net encoder was replaced by a residual encoder (3D_FullRes_ResEnc) [31]. The 3D_FullRes model utilized a six-stage PlainConvUNet architecture, trained with a patch size of 224 × 256 × 32 and a batch size of 2. The network incorporated progressively increasing feature channels (32 to 320) and employed anisotropic convolutional kernels (3 × 3 × 1) in the initial stage to account for lower through-plane resolution, followed by isotropic 3 × 3 × 3 kernels in subsequent layers. Downsampling was performed via strided convolutions, with two convolutional layers per stage, Instance Normalization and LeakyReLU activation. In the 3D_FullRes_ResEnc architecture, the encoder was replaced with residual blocks, incorporating varying numbers of convolutional blocks per stage (1, 3, 4, 6, 6, 6) to enhance feature representation, while maintaining the same decoder structure and training setup. All remaining architectural and training parameters were consistent with those described in the ovary AI model development section.

We trained 2 variants of the segmentation models. The model was trained to segment right and left testicles separately, as the development of each testicle has a significant value in fertility studies. This model was denoted as the Testicular_side-sep_ model. The second model was developed for segmenting testicles without identifying the right and left sides of the testicles separately. This model was denoted as the Testicular_Whole_ model. We trained this model to observe which one performs better and provides more robust results. So finally, 4 different models were trained, and performance was evaluated on the test set.

### 2.5. Model Evaluation and Statistical Analysis

Initially, the numbers of true positives (TP), false negatives (FN), and false positives (FP) of testicles, ovaries, and cysts segmentation were calculated. The performance of the AI models was evaluated using performance metrics including sensitivity, positive predictive value (PPV), and the Dice similarity coefficient (DSC). The DSC was calculated by considering the overlap between expert-annotated ground truth (GT) masks and AI-generated masks [28,29]. DSC was reported at the scan level for the ovary (DSC_ov_), cysts (DSC_CY_), and testicles (DSC_TS_) separately.

To understand the model’s clinical applicability, scan-level total ovary and cyst volumes and total testicle volumes were calculated for the AI-generated masks and expert-annotated GT masks. We calculated the total ovary volume (TOV), total cyst volume (TCV) using the following equations, for both the AI-generated mask and the GT mask:

(1) Total Ovary Volume = ∑(mask = 1)×S_x_ × S_y_ × S_z_ × 0.001

(2) Total Cyst Volume = ∑(mask = 2) × S_x_ × S_y_ × S_z_ × 0.001

where mask is the ovary and cyst contours with voxel values for ovary = 1, cyst = 2, and background = 0; S_x_, S_y_, S_z_ are the image spacing in the x, y, and z directions of the voxels of the mask in mm, and multiplied by 0.001 for conversion from mm^3^ to cm^3^.

We calculated the total testicular volume (TTV) using the following equation, for the AI-generated masks from both models and the GT mask:

(3) Total Testicular Volume = ∑(mask > 0) × S_x_ × S_y_ × S_z_ × 0.001

where mask is the testicles’ contours with voxel values greater than 0 (right testicle = 1, left testicle = 2 for the Testicular_side-sep_ model, and both testicles = 1 for the Testicular_Whole_ model), and the background voxel values are 0, S_x_, S_y_, and S_z_ are the image spacing in the x, y, and z directions of the voxels of the mask in mm, and multiplied by 0.001 for conversion from mm^3^ to cm^3^.

Further statistical analysis was performed to compare the TTV, TOV, and TCV from the AI-generated masks and GT masks. The Bland–Altman plot [32] of differences with mean difference (MD) and 95% confidence intervals (CI), and volume difference analysis per scan were analyzed. Given the intended use of the AI models in clinical research, whole-scan volumetric measurements were selected as the most appropriate metric for assessing clinical applicability. Additionally, the AI-predicted TOV and TTV across the whole female and male dataset were calculated per age group. Statistical analysis was performed using R Studio software (version 2023.12.0+369) and Python (version 3.10).

## 3. Results

### 3.1. Performance Evaluation of AI Models

#### 3.1.1. Ovary and Ovarian Cyst Segmentation AI

**Figure 3:**
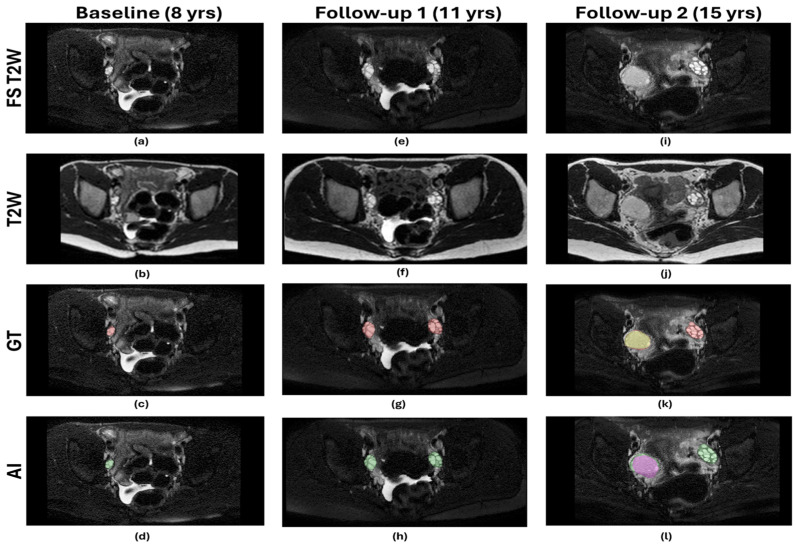
An example showing comparative results of the AI model with expert annotation, from a subject in the in-house test set, on different time points. The first column indicates the baseline scan of a girl at the age of 8 years. (**a**) FS T2W MRI, (**b**) T2W MRI, (**c**) expert-annotated ground truth (GT) of ovary (red), (**d**) AI-predicted ovary segmentation (green) with a DSC_OV_ = 0.87. The second column indicates a follow-up MRI at the age of 11 years, (**e**) FS T2W MRI, (**f**) T2W MRI, (**g**) expert-annotated ground truth (GT) of ovary (red), (**h**) AI-predicted ovary segmentation (green) with a DSC_OV_ = 0.94. For both time points, baseline and follow-up 1, the patient did not develop any cysts, and AI did not predict any cysts. The third column is MRI obtained at the age of 15 years, (**i**) FS T2W MRI, (**j**) T2W MRI, (**k**) expert-annotated ground truth (GT) of ovary (red) and cyst (yellow), (**l**) AI-predicted ovary segmentation (green) with DSC_OV_ = 0.80 and cyst segmentation (purple) with DSC_CY_ = 0.94. It is evident that, when a large ovarian cyst is present, the AI under-segments the ovarian volume.

**Figure 4:**
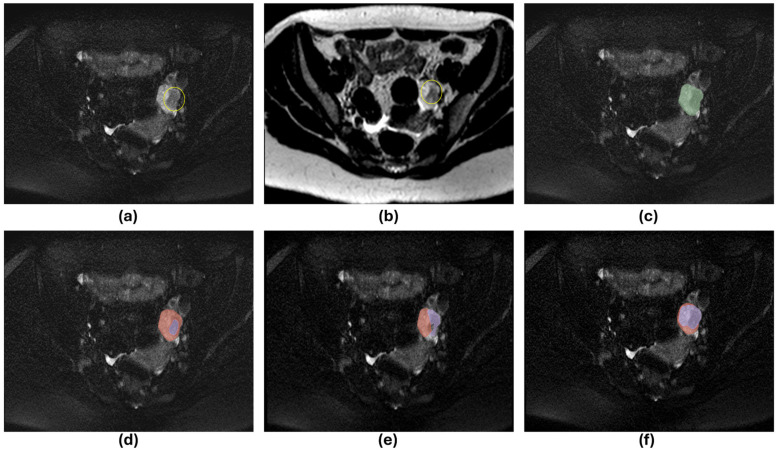
FS T2W and T2W MRI scans with FP cyst predicted by FS, T2W_FS_ + FS, and T2W + FS_T2W_ AI models on a subject from the in-house test cohort. (**a**) FS T2W MRI with visible cyst (yellow circle) with size <3 cm, (**b**) T2W MRI with visible cyst (yellow circle) with size <3 cm, (**c**) expert annotation on FS T2W MRI. The cyst was not contoured as the size of the cyst was less than 3 cm. (**d**) AI-predicted ovary (red) and cyst (purple) contours on the FS T2W scan by the T2W_FS_ + FS model. The T2W_FS_ + FS model is slightly under-segmenting the cyst. (**e**) AI-predicted ovary (red) and cyst (purple) contours on the FS T2W scan by the T2W + FS_T2W_ model. (**f**) AI-predicted ovary (red) and cyst (purple) contours on the FS T2W scan by the FS model. It is visible that the T2W + FS_T2W_ and FS models are over-segmenting the cysts and including the normal ovary region.

The AI-predicted masks generated by the models were compared with the expert-annotated GT masks to evaluate the model’s performance on the test. Only 14 scans contained cysts (4/4/6 for training/validation/testing, respectively). The performance of the models is presented in Table 2. The average DSC_OV_ of 0.89, 0.94, 0.92, 0.92, and DSC_CY_ of 0.96, 0.98, 0.97, 0.96 on the validation set were achieved by the T2W, FS, T2W_FS_ + FS, and T2W + FS_T2W_ models, respectively. The average DSC_OV_ of 0.75, 0.85, 0.86, 0.85, and DSC_CY_ of 0.85, 0.78, 0.69, 0.78 on the test set were achieved by the T2W, FS, T2W_FS_ + FS, and T2W + FS_T2W_ models, respectively. All the models detected each ovary and ovarian cyst present in the test set. The detection sensitivity for the ovary and ovarian cyst was thus 100% for all the models. The PPV of detecting the ovary was 100% for all models, with no FP detections of the ovary. PPVs of 100%, 87.5%, 100%, 77.78%, and 87.5% were found for the T2W, FS, T2W_FS_ + FS, and T2W + FS_T2W_ models, respectively. One, two, and one FP cysts were detected by the FS, T2W_FS_ + FS, and T2W + FS_T2W_ models. The T2W model did not have any FP cysts. Figure 3 shows AI prediction by the T2W_FS_ + FS model from three time point scans from a patient in the test set. Figure 4 shows the scan with an FP cyst identified by the three models (FS, T2W_FS_ + FS, and T2W + FS_T2W_ models). The one FP cyst detected by the three models using FS T2W MRI images (FS, T2W_FS_ + FS, and T2W + FS_T2W_ models) was a real cyst with a size <3 cm. As the expert-annotated cyst size was >3 cm, this cyst was not included during the annotation process due to the size cut-off for cysts. These apparent false positives reflect the model’s sensitivity to smaller, clinically plausible cysts that were excluded by the annotation criteria. Consequently, these detections were classified as false positives by all three models, despite representing true cysts.

Supplementary Figure S2 shows the Bland–Altman plot of the difference between ground truth masks and AI-predicted TOV and TCV for all the test participants. The values of the mean difference with a 95% confidence interval are demonstrated in Table 3 for all the models. The mean difference in TOV for all four models was above zero, indicating AI slightly under-segmented the ovary boundary. The mean difference for TCV is slightly below zero for the FS, T2W_FS_ + FS, and T2W + FS_T2W_ models, and slightly over zero for the T2W model. As the ovary and cyst contours were annotated on the FS T2W MRI, when we resampled the masks to T2W, in some cases, there can be slight misalignment due to the nature of the image acquisition, which can cause this difference for the T2W model. When a cyst is present, all the AI models are under-segmenting the ovary area. But for the scans without any cysts, the volume difference is very small (Figure S1). Based on all these analyses, it can be concluded that T2W_FS_ + FS would be more clinically applicable compared to the other models. AI-predicted TOV values per age group for this study are shown in Supplementary Figure S3a, and summary statistics values are reported in Supplementary Table S3.

#### 3.1.2. Testicle Segmentation AI

**Figure 5:**
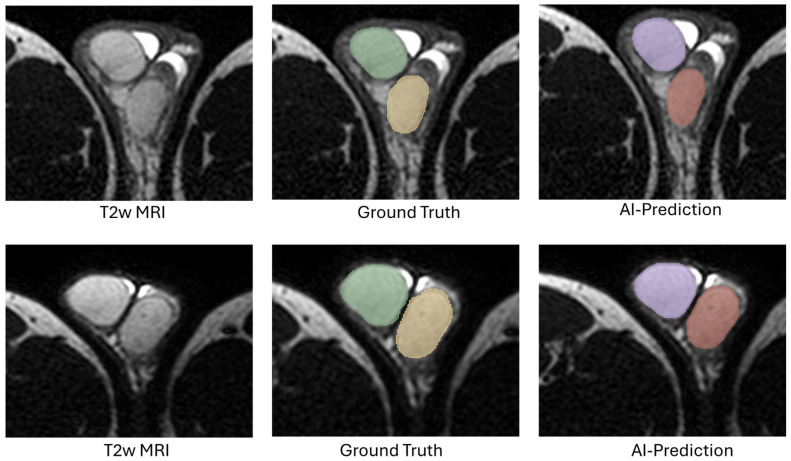
Example showing comparative results of the testicular AI models with expert annotation, from two subjects in the in-house test set. The first row is from a subject time scan from 2019 (age of16 years): (**a**) T2W MRI images, (**b**), expert-annotated ground truth (GT) with right (green) and left (yellow) testicles. (**c**) Right (purple) and left (red) testicles predicted by the Testicular_side-sep_ model with a Dice of 0.97, (**d**) both testicles (pink) predicted by the Testicular_Whole_ model with a Dice of 0.97. The second row is from a second subject time scan from 2022 (age of 15 years): (**e**) T2W MRI images, (**f**) expert-annotated ground truth (GT) with right (green) and left (yellow) testicles. (**g**) Right (purple) and left (red) testicles predicted by Testicular_side-sep_ model with dice of 0.96, (**h**) both testicles (pink) predicted by Testicular_Whole_ model with a Dice of 0.97.

The AI-predicted masks generated by the models were compared with the expert-annotated GT masks to evaluate the model’s performance on the test set. The performance of the models is presented in Table 4. Both 3d_FullRes and 3d_FullRes_ResEnc configurations for the Testicular_side-sep_ model achieved an average DSC of 0.90 on test sets. For the Testicular_side-sep_ model, the detection sensitivity for the right, left, and overall testicles was 94.92%, 100%, and 97.46% by 3d_FullRes and 96.61%, 100%, and 98.31% by 3d_FullRes_ResEnc configurations, respectively. For the Testicular_side-sep_ model, the PPV of detecting the right, left, and overall testicles was 98.25%, 100%, and 99.14% by 3d_Fullres and 98.28%, 100%, and 99.15% by 3d_FullRes_ResEnc configurations, respectively. Figure 5 shows the AI prediction by the 3d_FullRes and 3d_FullRes_ResEnc configuration models from two patients in the test set. Both of the configurations had one FP right testicles, which were from one of the scans with a retractile right testicle. Supplementary Figure S4 shows the scan with an FP testicle identified by 3D_FullRes configurations from a patient scan from 2014. Both of the configurations had FP for the same scan. This patient had a right retractile testicle, and both configurations identified a FP right testicle in the scrotum. Both configurations missed three right retractile testicles as FN. So, based on these results, the 3D_FullRes configuration was selected for the Testicular_side-sep_ model for further evaluation.

For the Testicular_Whole_ model, 3d_FullRes and 3d_FullRes_ResEnc configurations achieved an average DSC of 0.90 and 0.91 on test sets, respectively. For the Testicular_Whole_ model, the PPV of detecting the testicles was 97.35%, and 98.21% by 3d_FullRes and 3d_FullRes_ResEnc configurations, respectively. Both configurations missed three right testicles as FN, which were the three scans with a retractile right testicle, which we expected the model to ignore. The 3d_FullRes and 3d_FullRes_ResEnc configurations detected three and two FP testicles, respectively. Supplementary Figure S4 shows the two FP testicles identified by both configurations from the same patients’ scans from 2015 and 2017 (age 8, 10), but for these scans, AI predicted both testicles accurately. So, based on these results, the 3d_FullRes_ResEnc configuration was selected for the Testicular_Whole_ model, for further evaluation.

False positives predominantly arose in cases with retractile testes across both model configurations, where variability in anatomical position led to misidentification of the testicle within the scrotum. As the models were not trained on cases with retractile testes, they were not conditioned to account for the absence of the testicle in the scrotum and therefore incorrectly identified the scrotal region as containing a testicle, resulting in false positives.

**Figure 6:**
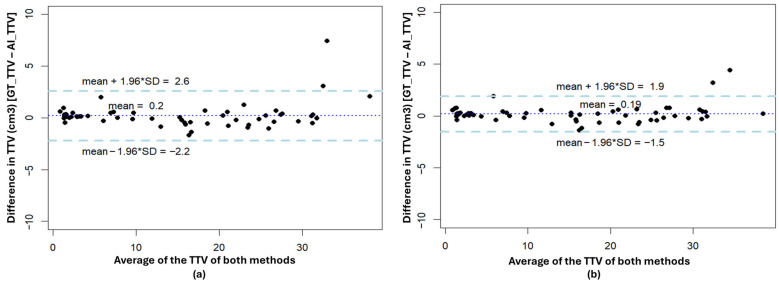
Bland–Altman (BA) plot of difference in TTV (cm^3^) between expert annotation GT and AI predicted Testicles masks by (**a**) Testicular_side-sep_ and (**b**) Testicular_Whole_ models from the in-house test cohort.

Figure 6 shows the Bland–Altman plot of the difference between GT masks and AI-predicted TTV for all the test scans for the Testicular_side-sep_ and Testicular_Whole_ models. The mean difference in TTV between AI and expert was very small, 0.2 cm^3^ and 0.19 cm^3^ for the Testicular_side-sep_ and Testicular_Whole_ models, respectively. AI model results for two scans were outside the 95% confidence interval due to a high-volume difference. Supplementary Figure S5A,B show the difference in the testicular boundary by the Testicular_Whole_ model in comparison to the expert annotation. Supplementary Figure S5C,D show that the Testicular_side-sep_ model predicted left testicles of these scans as both right and left testicles. We have written a Python code to fix the AI prediction automatically as a post-processing step, based on connected-component analysis. Based on all these analyses, it can be concluded that both Testicular_side-sep_ and Testicular_Whole_ models showed similar performance and potential clinical applicability. AI-predicted TTV values per age group for this study are shown in Supplementary Figure S3b, and summary statistics values are reported in Supplementary Table S4.

## 4. Discussion

Gonadal development plays a critical role during adolescence and has lasting effects on human fertility. Although monitoring gonadal growth is essential for studies related to puberty, fertility, and adolescent development, MRI remains underutilized as a quantitative assessment tool in this context. In this study, we developed an automated, AI-based approach to assess gonadal growth in both boys and girls using MRI. Our best-performing AI models achieved DSC of 0.86, 0.69, and 0.90, and sensitivities of 100%, 100%, and 98.31% for the segmentation and detection of ovaries, ovarian cysts in girls, and testicles in boys, respectively. Because our model was developed using data from boys and girls transitioning from the pre-pubertal phase to puberty and adulthood, it is suitable for application in both young and adult subjects.

In clinical research, MRI plays a crucial role for the evaluation of gonadal health. T2W MRI offers valuable anatomical information, while FS T2W MRI enhances the visibility of internal structures and pathologies within the gonads by providing additional functional information [13,33]. Because ovarian cysts and follicles are fluid-filled structures, they appear with hyperintense signal on FS T2W MRI, facilitating their relatively straightforward detection [34]. In contrast, T2W MRI provides more detailed structural information about the ovary [16]. Therefore, we investigated both sequences individually and in combination for training our AI model. Our findings indicate that the model trained on the combined T2W and FS T2W MRI sequences achieved the best performance. Alternatively, as testicular anatomy is clearly visualized on T2W MRI alone [13], we utilized only this single sequence for testicular segmentation.

Due to the scarcity of annotated MRI datasets, limited literature is available on gonad segmentation using MRI. None of the current organ segmentation AI models, i.e., TotalSegmentator [19], VISTA3D [35], MRISegmenter [36], or organ segmentation foundation models like MEDSAM [37], provides gonad segmentations. A recent study by Zeng et al. used the Segment Anything Model 2 (SAM 2) extension from the 3D slicer tool (www.slicer.org) to segment ovarian follicles from US and MRI and showed that 3D MRI performs better in segmenting follicles to diagnose polycystic ovary syndrome (PCOS) [38]. However, they used a semi-automatic process, which can be time-consuming and prone to reader biases. Sun et al. [39] developed a ResUNet-based AI model using T2W MRI from 160 subjects (mean age 35  ±  15 years) to segment right and left testicles and achieved a Dice of 0.926  ±  0.034. However, their model had an average volume difference of 0.815  ±  0.824 cm^3^ when comparing manual and automated measurements. In comparison, our model achieved a more reliable mean volume difference of 0.2 ± 1.2 cm^3^. Currently, there are no studies of pre-pubertal healthy subjects using AI methodology to segment developing, difficult-to-identify gonads. Both of our models showed reliable performance in both pre-pubertal and post-pubertal subjects.

This proof-of-concept study demonstrates the feasibility of automated gonadal volume detection in both pre- and post-pubertal populations, highlighting its potential for broader application in clinical practice and research settings, with further validation on larger cohorts warranted. Specifically, these models facilitate the automated extraction of gonadal volume measurements in longitudinal research cohorts, enable the tracking of growth trajectories and gonadal development, promote studies exploring the neuroendocrine regulation of puberty, and support the identification of structural biomarkers from MRI data relevant to pubertal development. Furthermore, our AI models may aid in the early diagnosis of delayed puberty, hypogonadism, endocrine disorders, gonadal dysgenesis, and fertility disorders, including the development of polycystic ovarian syndrome, ovarian hyperstimulation syndrome, and testicular dysgenesis syndrome. Notably, approximately 20% of women develop ovarian cysts over their lifetime, which are generally benign, asymptomatic, and infrequently require intervention [40]. Nevertheless, complications such as pelvic pain, cyst rupture, hemorrhage, or ovarian torsion can occasionally arise, necessitating timely clinical management [41]. Therefore, reliable detection of ovarian cysts during routine surveillance can be crucial. Our model exhibited promising performance in identifying ovarian cysts across both young and young adult cohorts, suggesting its utility for clinicians in routine practice. Additionally, the testicular model may also assist in identifying individuals with retractile or undescended testicles and enable investigation into risk factors associated with cryptorchidism. To facilitate further research, we have made the model weights publicly available, thereby supporting the broader scientific community in puberty and fertility studies. In the future, the analysis of large MRI datasets using these models may enable the establishment of normative gonadal growth curves and the identification of fertility-related structural benchmarks across diverse populations.

Our study has some limitations. First, both models were trained on a relatively small sample size from a single institution, emphasizing the need for external validation using larger, multicenter cohorts. Additionally, the ovary and cyst segmentation model included very few cases with physiological cysts. Due to the limited availability of subjects, our study focused only on cysts larger than 3 cm; although the model was able to detect cysts smaller than 3 cm, further investigation is required to assess its performance in routinely identifying smaller cysts (<3 cm). Due to the small sample size, the models were trained on only one-fold; further studies on a larger cohort are necessary for multi-fold cross-validation model development. Moreover, the testicular model is currently limited to the segmentation of the healthy testicles, and none of the subjects in the present cohort exhibited testicular masses. Future work will aim to expand the cohort to include cases with detecting testicular masses for improved model robustness. Furthermore, our ground-truth annotations were generated by a single expert radiologist. Inter-observer and intra-observer agreement assessments were not performed and will be considered in future work. Finally, AI-based measurements were not correlated with clinical or laboratory data in this study; however, we plan to address this limitation in the subsequent research.

## 5. Conclusions

In conclusion, we have developed two open-source AI tools to automatically segment and quantify gonadal volumes using MRI data from healthy pre-, pubertal, and post-pubertal boys and girls. The developed models exhibit promising and reliable performance in volumetric and morphologic evaluation of gonads during puberty.

## Figures and Tables

**Table 1:** Image acquisition parameters of the clinical cohort with median (min, max) values.

	Girls (*n* = 44)		Boys (*n* = 88)
Modality	*T2W*	*FS T2W*	*T2W*
No of Scans	*15 (6, 26)*	*11 (5, 22)*	*6 (1, 12)*
Phase Field of View (%)	100 (81.08, 100)	100 (80.82, 100)	100 (80, 100)
Acquisition matrix	160 × 160 (120 × 119, 180 × 179)	320 × 320 (240 × 240, 360 × 359)	160 × 160 (140 × 140, 180 × 177)
Repetition time (msec)	1600	1600 (1600, 1753.23)	1600
Echo time (msec)	120	120	120
Flip angle (degrees)	90	90	90
Pixel Spacing (mm)	0.6 (0.52, 0.6)	0.75 (0.47, 0.75)	0.6 (0.56, 0.75)
Slice thickness (mm)	2 (2 [193 scans], 4 [ 1 scan])	2	2 (2 [486 scans], 4 [5 scans])
spacing between slices	2.2 (2.2 [*n* = 193 scans], 5 [*n*= 1 scans])	2.2	2.2 (2.2 [486 scans], 5 [5 scans])
Image shape (pixels)	400 × 400 (320 × 320, 480 × 480)	320 × 320 (320 × 320, 480 × 480)	400 × 400 (320 × 320, 480 × 480)
Reconstruction Diameter (mm)	240 (180, 270)	240 (180, 270)	240 (210, 270)
Time for acquisition (min:sec)	1:36 (0:48, 2:08)	1:36 (1:36, 3:12)	1:36 (0:48, 3:00)
Echo train length	51 (49, 84)	134 (76, 138)	51 (51, 104)
Number of Averages	1 (1, 2)	1 (1, 2)	1 (1, 3)
Manufacturer	Philips Medical Systems	Philips Medical Systems	Philips Medical Systems
Model Name	Achieva	Achieva	Achieva

**Table 2:** Ovary and ovarian cyst segmentation models performance on the in-house test cohort.

Organ/Cyst Level	T2W		FS		T2W_FS_ + FS		T2W + FS_T2W_	
	Ovary	Cyst	Ovary	Cyst	Ovary	Cyst	Ovary	Cyst
TP	72	7	72	7	72	7	72	7
FN	0	0	0	0	0	0	0	0
FP	0	0	0	1	0	2	0	1
Sensitivity	100	100	100	100	100	100	100	100
PPV	100	100	100	87.5	100	77.78	100	87.5
Average Dice (min, max)	0.75 (0.28, 0.90)	0.85 (0.51, 0.95)	0.85 (0.53, 0.94)	0.78 (0, 0.96)	0.86 (0.63, 0.94)	0.69 (0, 0.96)	0.86 (0.58, 0.93)	0.78 (0, 0.96)

**Table 3:** Average of mean difference with 95% CI (cm^3^) between AI-predicted and GT TOV and TCV on the in-house test cohort.

	T2W	FS	T2WFS + FS	T2W + FST2W
TOV	2.4 (−8.8, 14)	1.3 (−5.9, 8.5)	0.87 (−5.78, 7.5)	−1.21 (−6.0, 8.4)
TCV	0.39 (−3.6, 4.4)	−0.56 (−4.4, 3.3)	−0.41 (−3.3, 2.5)	0.46 (−4.3, 3.3)

**Table 4:** Testicle segmentation models performance on the in-house test cohort.

Summary	Testicular_side-sep_ Model						Testicular_Whole_ Model	
	3d_FullRes			3d_FullRes_ResEnc			3d_FullRes	3d_FullRes_ResEnc
	Right Testicle	Left Testicle	Overall	Right Testicle	Left Testicle	Overall	Overall	Overall
TP	56	59	115	57	59	116	115	115
FN	3	0	3	2	0	2	3	3
FP	1	0	1	1	0	1	3	2
Sensitivity	94.92	100	97.46	96.61	100	98.31	97.35	97.35
PPV	98.25	100	99.14	98.28	100	99.15	97.35	98.21
Average Dice (min, max)	0.90 (0.52, 0.97)			0.90 (0.49, 0.97)			0.90 (0.52, 0.97)	0.91 (0.54, 0.97)

## Data Availability

Some or all datasets generated during and/or analyzed during the current study are not publicly available but are available from the corresponding author on reasonable request. The models, checkpoints, and codes, with a few examples to replicate the results, are publicly available at: https://github.com/HFahmida/MRI-Gonad_SegAI.git.

## Supplementary Materials

The following supporting information can be downloaded at: https://www.mdpi.com/article/10.3390/diagnostics16091357/s1, Table S1: Data split for ovary and ovarian cyst segmentation AI development. Table S2: Data split for Testicle segmentation AI development. Table S3: Summary Statistics of AI quantified total ovary volume for all in-house female subjects per age group. Table S4: Summary Statistics of AI quantified total testicular volume for all in-house male subjects per age group. Figure S1: Flowchart of inclusion and exclusion criteria for the (a) female subject selection for ovary and ovarian cyst segmentation AI development, (b) male subject selection for testicular segmentation AI development. Figure S2: Bland Altman (BA) plot of difference in Total Ovarian Volume, TOV (row 1) and Total Cyst Volume, TCV (row 2) [cm^3^] between expert annotation Ground Truth (GT) and AI predicted ovary and cyst masks by models using axial and sagittal T2 turbo spin echo (TSE) and axial fat-saturated (FS) weighted MRI scan data. T2-weighted (T2W) MRIs were resampled to the corresponding FS T2W MRI size and spacing and denoted as T2W_FS_, and similarly, FS T2W MRI and expert-annotated masks were resampled to T2 space and spacing and denoted as FS_T2W_. The first row shows the BA plot for the difference in TOV between GT and AI prediction by (a) T2w_FS_+FS, (b) FS_T2w_+T2w, (c) T2w, and (d) FS models. The second row shows the BA plot for the difference in TCV between GT and AI prediction by (a) T2w_FS_+FS, (b) FS_T2w_+T2w, (c) T2w, and (d) FS models. Figure S3: (a) AI predicted Total ovary volumes across different age groups in the female dataset. (b) AI predicted total testicular volumes across different age groups in the male dataset. Figure S4: (A) T2W image and False Positive (FP) testicles prediction (red) by Testicular_side-sep_ model from a patient scan from 2014 (age 8). (B) T2W image and FP testicle prediction (red) by Testicular_Whole_ model from a patient scan from 2015 (age 8). (B) T2W image and FP testicle prediction (red) by Testicular_Whole_ model from the same patient as B, scan from 2017 (age 10). Figure S5: (A) T2W image and testicular annotation by expert Ground Truth (GT; green) and Testicular_Whole_ model prediction (red) from a patient scan from 2021 (age 17) with total testicular volume difference of 3.2 cm^3^. (B) T2W image and testicular annotation by expert GT (green) and Testicular_Whole_ model prediction (red) from a patient scan from 2021 (age 15) with total testicular volume difference of 4.4 cm^3^. (C) T2W image and right (green) and left (yellow) testicles prediction by Testicular_side-sep_ model from a patient scan from 2016 (age 13) with total testicular volume difference of 3.07 cm^3^. (D) T2W image and right (green) and left (yellow) testicles prediction by Testicular_side-sep_ model from a patient scan from 2022 (age 16) with total testicular volume difference of 2.05 cm^3^. For (C) and (D), it is evident that, Testicular_side-sep_ model has partially detected left testicles (yellow) as right testicles (green).

## Abbreviations

The following abbreviations are used in this manuscript:

AI	Artificial Intelligence
MRI	Magnetic Resonance Imaging
T2W	T2-Weighted
T1W	T1-Weighted
FS	Fat-Saturated
US	Ultrasound
PCOS	Polycystic Ovary Syndrome
TSE	T2 Turbo Spin Echo
GT	Ground Truth
T2WFS	T2W MRIs Resampled to FS T2W MRI
FST2W	FS T2W MRI Resampled to T2W MRI
3D-FullRes	3D Full-Resolution nnUnet Configuration
3d_FullRes_ResEnc	3D Full-Resolution nnUnet Configuration with a Residual Encoder
TP	True Positives
FN	False Negatives
FP	False Positives
PPV	Positive Predictive Value
DSC	Dice Similarity Coefficient
DSCov	DSC for Ovaries
DSCCY	DSC for Ovarian Cysts
DSCTS	DSC for Testicles
TOV	Total Ovary Volume
TCV	Total Cyst Volume
TTV	Total Testicular Volume
MD	Mean Difference
CI	Confidence Interval
