# Supplementary material for: Artificial Intelligence-Based Approach for Automated Gonad Volume Quantification Using Magnetic Resonance Imaging in Healthy Adolescents Across Puberty
Source: Diagnostics (Basel). 2026 Apr 30;16(9):1357. doi: 10.3390/diagnostics16091357 (PMC13163603; doi:10.3390/diagnostics16091357)
Supplement: Supplementary file 1 [file diagnostics-16-01357-s001.zip › diagnostics-4220546-supplementary.pdf]

Artificial Intelligence-Based Approach for Automated Gonad Volume Quantification Using Magnetic Resonance Imaging in Healthy Adolescents Across Puberty

Fahmida Haque [1,2], Stephanie A. Harmon [1,2], Allison Kumnick [3], Mary Soliman [3], Karen F. Berman [4], Jack A. Yanovski [5], Evrim B. Turkbey [6], Lynnette K. Nieman [7], Veronica Gomez-Lobo [3], Shau-Ming Wei [8], Peter J. Schmidt [8] and Baris Turkbey [1,2,*]

Supplementary Tables:

Supplementary Table S1: Data split for ovary and ovarian cyst segmentation AI development

Data splits	*Subjects*	*Scans with ovary annotation*	*Scans with Cysts (>3cm)*
**Train**	14	123	4
**Validation**	3	15	4
**Test**	5	32	6

Supplementary Table S2: Data split for Testicle segmentation AI development

	**Subjects**	**Total scans**	**Total scans at age 8**	**Total scans at age 9**	**Total scans at age 10**	**Total scans at age 11**	**Total scans at age 12**	**Total scans at age 13**	**Total scans at age 14**	**Total scans at age 15**	**Total scans at age 16**	**Total scans at age 17**
Total	44	266	22	24	26	24	30	34	26	35	25	20
Train	30	180	16	16	16	17	21	21	18	23	18	14
Test	9	59	4	6	7	4	6	9	6	8	5	4
Validation	5	27	2	2	3	3	3	4	2	4	2	2

Supplementary Table S3: Summary Statistics of AI quantified total ovary volume for all in-house female subjects per age group

Age Group (years)	n	mean	sd	median	q1	q3	min	max
8	47	1.98	1.41	1.75	1.10	2.25	0.41	7.06
9	52	3.37	2.25	2.95	1.91	3.73	0.59	10.04
10	43	4.56	2.22	4.01	3.15	6.25	0.69	10.43
11	37	5.98	2.84	5.49	4.22	7.27	1.06	12.22
12	56	9.48	7.52	7.77	6.12	9.94	2.34	52.83
13	55	10.71	4.47	10.29	8.30	12.35	2.89	25.90
14	45	12.70	7.97	11.07	7.35	14.50	3.05	50.19
15	39	16.77	20.39	11.77	7.98	15.15	4.35	117.75
16	32	17.73	12.26	13.60	9.14	21.86	7.81	59.92
17	22	16.41	13.42	12.19	8.16	19.68	3.97	56.90

Supplementary Table S4: Summary Statistics of AI quantified total testicular volume for all in-house male subjects per age group

Age Group (years)	n	mean	sd	median	q1	q3	min	max
8	60	1.93	0.59	2.01	1.65	2.24	0.58	4.02
9	55	2.20	0.85	2.10	1.61	2.56	0.83	5.61
10	51	3.78	2.88	2.68	2.13	4.66	1.06	13.26
11	49	7.15	6.86	4.23	2.70	9.40	1.70	35.10
12	57	12.39	7.57	10.49	6.08	17.36	2.30	29.55
13	64	20.44	7.48	21.44	17.08	25.57	3.48	36.29
14	44	24.28	8.36	25.68	19.58	29.75	3.80	36.38
15	51	29.96	8.06	29.31	25.23	36.74	11.93	46.53
16	37	33.33	8.08	33.08	29.93	38.56	16.11	51.27
17	31	33.30	9.44	33.53	29.67	40.02	12.52	54.18

Supplementary Figures:

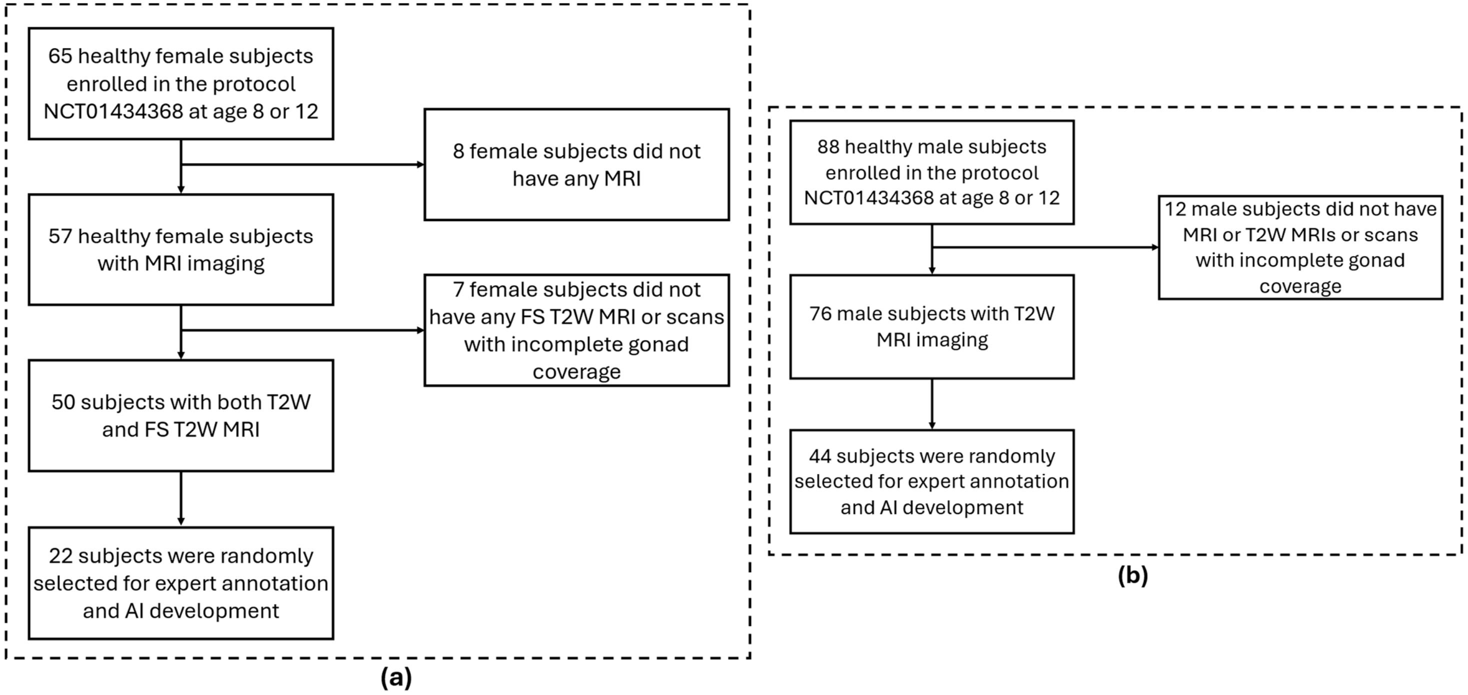

Supplementary Figure S1: Flowchart of inclusion and exclusion criteria for the (a) female subject selection for ovary and ovarian cyst segmentation AI development, (b) male subject selection for testicular segmentation AI development.

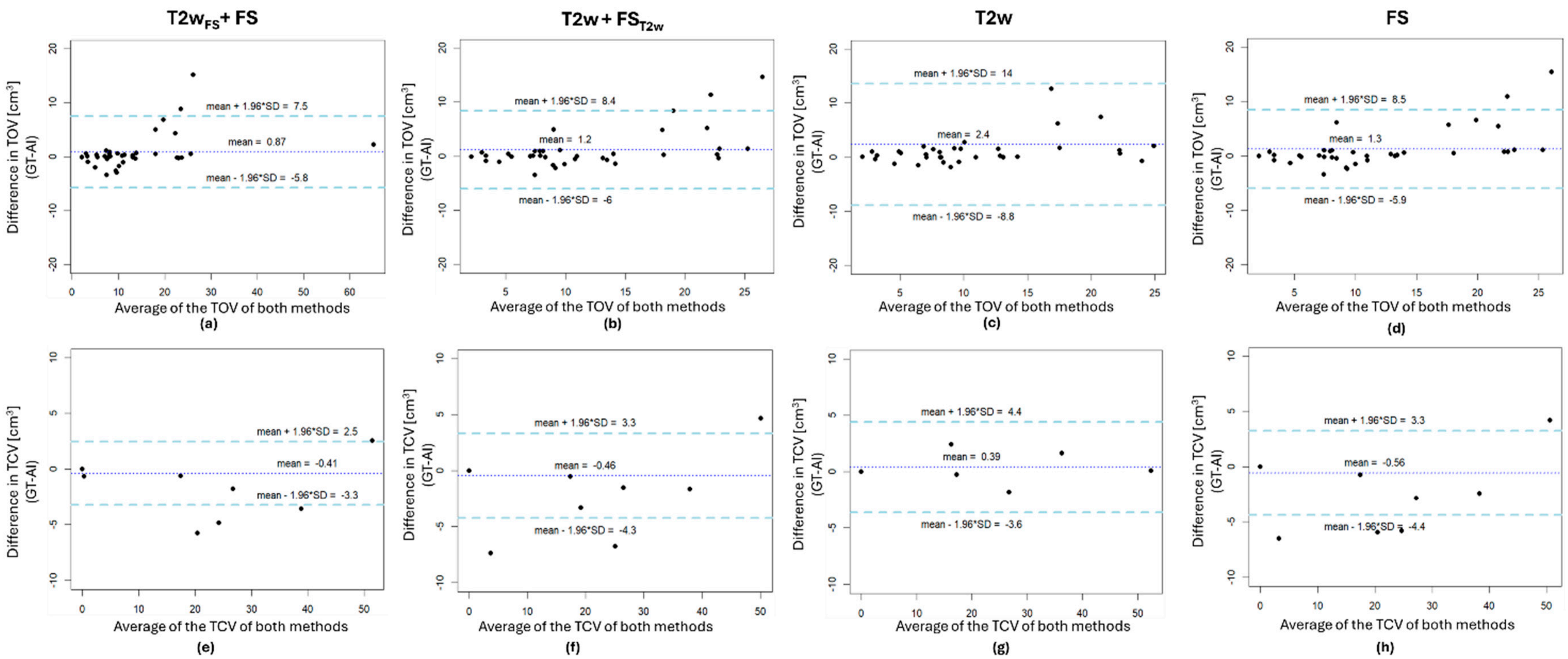

Supplementary Figure S2: Bland Altman (BA) plot of difference in Total Ovarian Volume, TOV (row 1) and Total Cyst Volume, TCV (row 2) [cm^3] between expert annotation Ground Truth (GT) and AI predicted ovary and cyst masks by models using axial and sagittal T2 turbo spin echo (TSE) and axial fat-saturated (FS) weighted MRI scan data. T2-weighted (T2W) MRIs were resampled to the corresponding FS T2W MRI size and spacing and denoted as $T2W_{FS}$, and similarly, FS T2W MRI and expert-annotated masks were resampled to T2 space and spacing and denoted as FS_{T2W}. The first row shows the BA plot for the difference in TOV between GT and AI prediction by (a) $T2w_{FS}$+FS, (b) FS_{T2w}+T2w, (c) T2w, and (d) FS models. The second row shows the BA plot for the difference in TCV between GT and AI prediction by (a) $T2w_{FS}$+FS, (b) FS_{T2w}+T2w, (c) T2w, and (d) FS models.

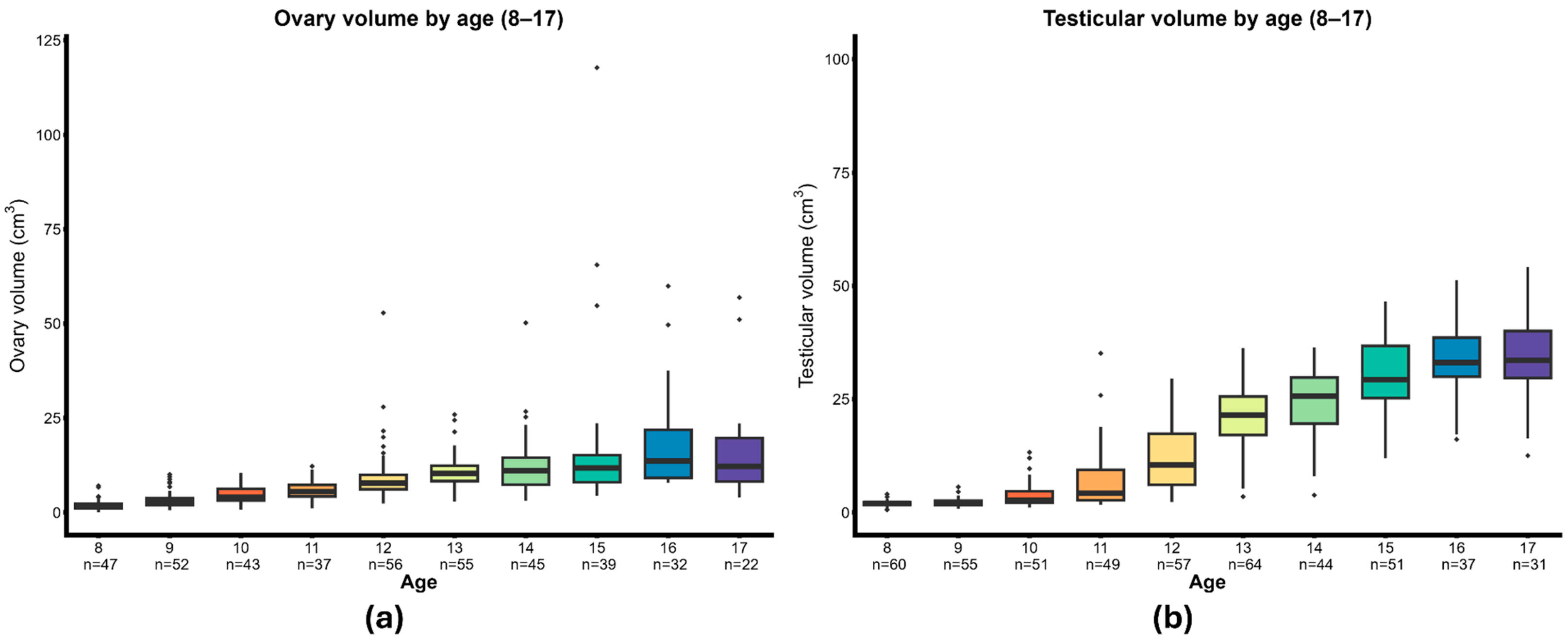

Supplementary Figure S3: (a) AI predicted Total ovary volumes across different age groups in the female dataset. (b) AI predicted total testicular volumes across different age groups in the male dataset.

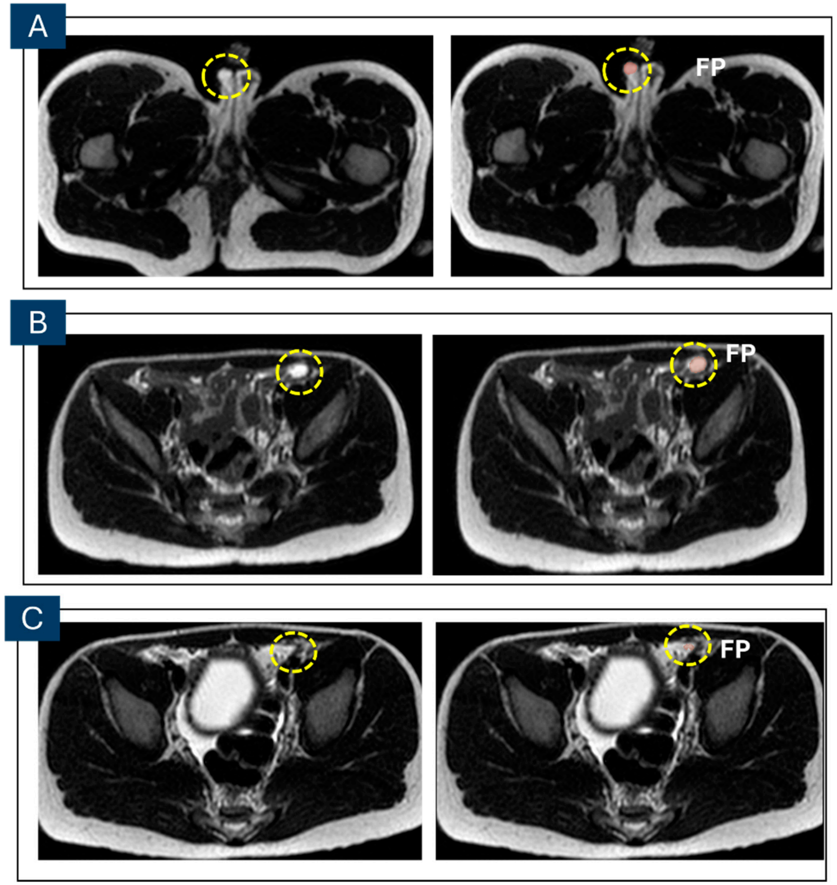

Supplementary Figure S4: (A) T2W image and False Positive (FP) testicles prediction (red) by $Testicular_{side\text{-}sep}$ model from a patient scan from 2014 (age 8). (B) T2W image and FP testicle prediction (red) by $Testicular_{Whole}$ model from a patient scan from 2015 (age 8). (B) T2W image and FP testicle prediction (red) by $Testicular_{Whole}$ model from the same patient as B, scan from 2017 (age 10).

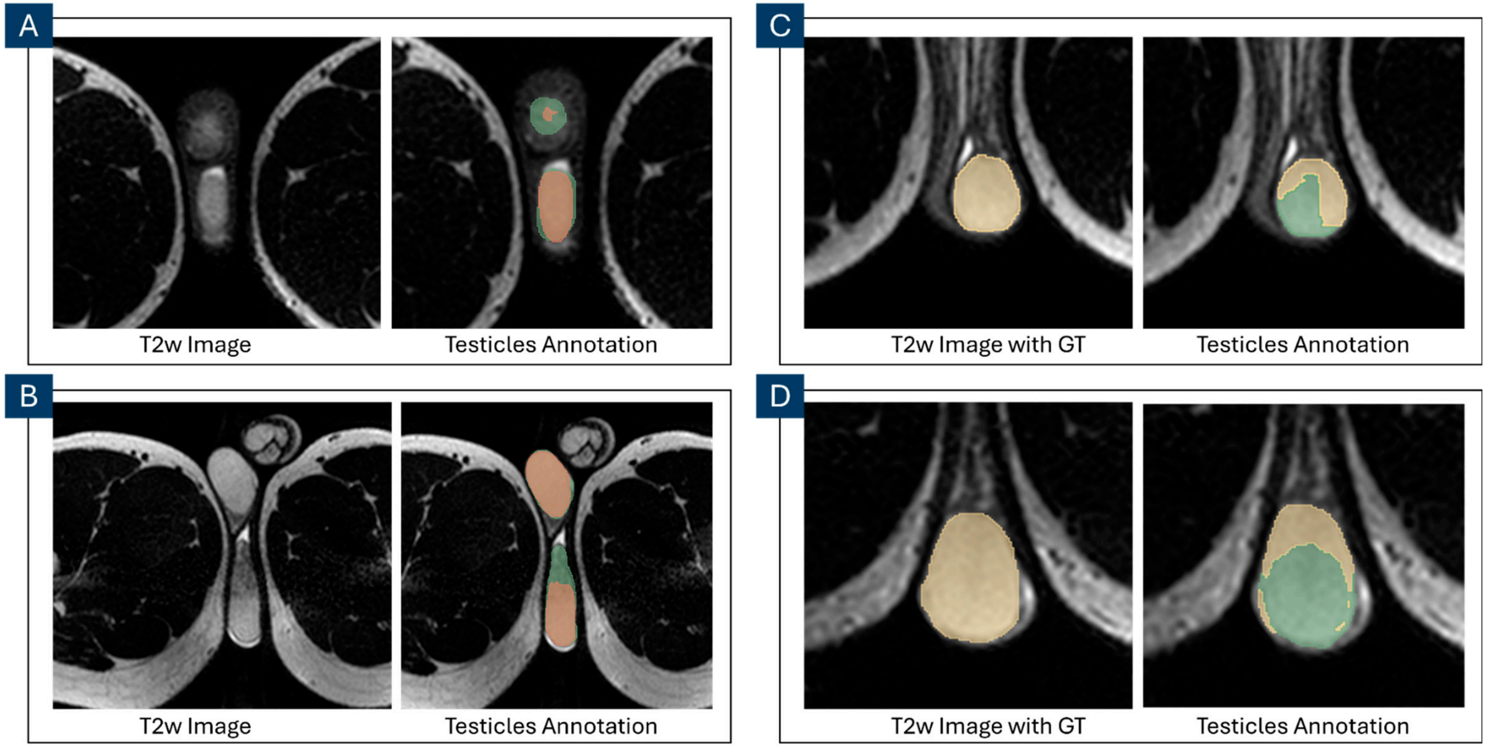

Supplementary Figure S5: (A) T2W image and testicular annotation by expert Ground Truth (GT; green) and $Testicular_{Whole}$ model prediction (red) from a patient scan from 2021(age 17) with total testicular volume difference of 3.2 cm3. (B) T2W image and testicular annotation by expert GT (green) and $Testicular_{Whole}$ model prediction (red) from a patient scan from 2021 (age 15) with total testicular volume difference of 4.4 cm^3. (C) T2W image and right (green) and left (yellow) testicles prediction by $Testicular_{side\text{-}sep}$ model from a patient scan from 2016 (age 13) with total testicular volume difference of 3.07 cm3. (D) T2W image and right (green) and left (yellow) testicles prediction by $Testicular_{side\text{-}sep}$ model from a patient scan from 2022 (age 16) with total testicular volume difference of 2.05 cm3. For (C) and (D), it is evident that, $Testicular_{side\text{-}sep}$ model has partially detected left testicles (yellow) as right testicles (green).
